# *In Vivo* Motility Patterns Displayed by Immune Cells Under Inflammatory Conditions

**DOI:** 10.3389/fimmu.2021.804159

**Published:** 2022-01-03

**Authors:** Diego Ulisse Pizzagalli, Alain Pulfer, Marcus Thelen, Rolf Krause, Santiago F. Gonzalez

**Affiliations:** ^1^ Istituto di Ricerca in Biomedicina (IRB), Università della Svizzera italiana, Bellinzona, Switzerland; ^2^ Euler institute, Università della Svizzera italiana, Lugano-Viganello, Switzerland; ^3^ Department of Information Technology and Electrical Engineering, Swiss Federal Institute of Technology Zurich (ETHZ) Zürich, Zürich, Switzerland

**Keywords:** cell actions, computer vision, inflammation, intravital imaging, leukocytes, motility patterns

## Abstract

The migration of immune cells plays a key role in inflammation. This is evident in the fact that inflammatory stimuli elicit a broad range of migration patterns in immune cells. Since these patterns are pivotal for initiating the immune response, their dysregulation is associated with life-threatening conditions including organ failure, chronic inflammation, autoimmunity, and cancer, amongst others. Over the last two decades, thanks to advancements in the intravital microscopy technology, it has become possible to visualize cell migration in living organisms with unprecedented resolution, helping to deconstruct hitherto unexplored aspects of the immune response associated with the dynamism of cells. However, a comprehensive classification of the main motility patterns of immune cells observed *in vivo*, along with their relevance to the inflammatory process, is still lacking. In this review we defined cell actions as motility patterns displayed by immune cells, which are associated with a specific role during the immune response. In this regard, we summarize the main actions performed by immune cells during intravital microscopy studies. For each of these actions, we provide a consensus name, a definition based on morphodynamic properties, and the biological contexts in which it was reported. Moreover, we provide an overview of the computational methods that were employed for the quantification, fostering an interdisciplinary approach to study the immune system from imaging data.

## Introduction

Inflammation is a highly dynamic process that involves changes in cell behavior both at the site of the insult as well as at distant organs (1, 2). Immune cells are key players in this process, as they relocate into inflamed tissues, and secrete mediators of inflammation that orchestrate a cascade of immune reactions (3–7).

Over the last two decades, intravital microscopy (MP-IVM) techniques have consolidated the *in vivo* analysis of the immune response. Videos acquired *via* MP-IVM capture the behavior of immune cells, including their migratory and interaction patterns, in organs of living organisms (8–11). However, the quantification of these videos remains challenging. This is due to a range of factors, such as the complexity of the *in vivo* environment, which includes a multitude of cell types and anatomical structures (12) or the high plasticity and dynamism of the migration patterns displayed by immune cells, which change over time. Moreover, numerous technical artifacts introduced by the intravital imaging procedure also affect the analysis to a large extent (13).

The recently established image-based systems biology paradigm offers a unique opportunity to study cell behavior *in vivo*, as it combines imaging data and computational methods (14). Analogously, recently developed computer vision methods for action recognition (AR) have enabled the analysis of the complex behavior of humans associated with specific actions such as walking, jumping, etc. (15). This is a particularly challenging task, as human actions may be hierarchical in their nature, composed of multiple actors, or captured by different imaging modalities (15). Interestingly, these three challenges are shared with the quantification of the immune cell behavior in intravital movies, as they display different morphodynamics, undergo cell-to-cell interactions, and can be imaged in different anatomical regions. Hence, in line with AR, we define cell actions as motility patterns associated with relevant biological functions to dissect leukocyte behavior.

To this end, we collected and reported from the literature a list of actions displayed by immune cells in different organs during key inflammatory processes. A summary of the diseases, organs, and studies included in this review is reported in **Table 1**. Moreover, we provide a consensus definition for each action and its biological relevance during inflammation. Lastly, we report the computational methods currently available for the detection and quantification of each reviewed action.

**Table 1:** Summary of the actions described in different inflammatory conditions, organs, and cell types.

Condition	Organ	Cell type	Reported actions
**Acute inflammation**	Kidney	Monocytes	Patrolling (16)
		Monocytes Neutrophils	Contact formation (16)
**Chronic inflammation**	Liver	NKT	Directed (17) Patrolling (17) Swarming (17)
**Hypersensitivity**	Lymphatics	T	Arrested (18) Patrolling (18) Swarming (18)
		T DCs	Contact formation (18)
		DCs	Arrested (18) Patrolling (18)
**Induced/Sterile inflammation**	Vasculature	Monocytes	Patrolling (19–21)
		Neutrophils	Directed (22) Arresting (22)
	LN	NK	Contact formation (23) Patrolling (23, 24)
		T cells	Contact formation (23)
		NKs B	Contact formation (24)
		B	Arrested (25)
		T	Arrested (26) Patrolling (27)
		T DCs	Contact formation (27–31)
	Skin	Neutrophils	Directed (32) Swarming (32)
	Lung	Eosinophils	Directed (33)
	Kidney	Monocytes	Patrolling (16)
	CNS	Monocytes	Patrolling (34)
	Liver	Neutrophils	Directed (35) Swarming (35)
**Infection**	Spleen	Neutrophils	Directed (36) Patrolling (36)
		DCs	Swarming (36)
		T	Arrested (36)
		Monocytes	Swarming (36)
	Skin	Neutrophils	Directed (37) Swarming (38, 39)
		Eosinophils Macrophages	Contact formation (40)
		Eosinophils	Arrested (40) Directed (40)
	Lung	Neutrophils	Patrolling (38) Swarming (38)
	LN	Neutrophils	Arrested (41) Directed (41) Swarming (42)
		NKs DCs	Contact formation (43)
		NKs	Arrested (43)
**Injury**	Skin	Neutrophils	Directed (44, 45) Swarming (44, 45)
**Steady state**	Vasculature	Monocytes	Patrolling (19, 20)
	Skin	Eosinophils	Patrolling (40)
		Eosinophils Macrophages	Contact formation (40)
	LN	T	Patrolling (46, 47)
	Lung	Eosinophils	Patrolling (33)
**Tumor**	Lungs	Monocytes	Patrolling (49)
	Ovary	T	Directed (50)
		
**Vaccination**	LN	Neutrophils	Arrested (51) Directed (51) Patrolling (51) Swarming (51)
	Vasculature	Monocytes	Patrolling (52)

## Intravital Imaging Workflow

The application of MP-IVM for the imaging of multiple cells during the inflammatory process involves the following steps.

### Cell labeling

Different methods are available, including the adoptive transfer of cells from transgenic animals expressing a fluorophore-tagged protein, *in vitro* labeling with fluorescent dies, or the injection of fluorescently labeled antibodies that specifically bind to the cells of interest. Available optical probes for MP-IVM and fluorescent proteins are comprehensively reviewed elsewhere (53, 54) and are beyond the scope of this work.

### Surgery

**Figure 1:**
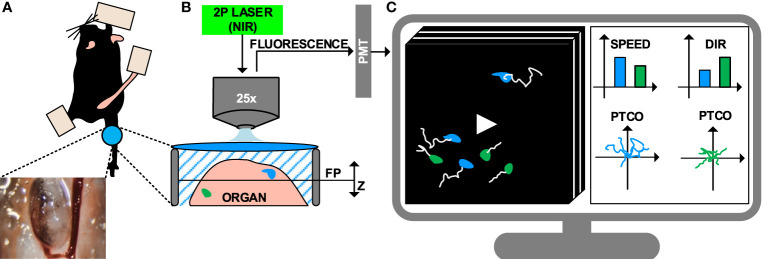
Intravital imaging of the immune system under inflammatory conditions. **(A)** Representation of the surgical model used to perform intravital imaging in the murine popliteal lymph node, including a minimally invasive surgery and imaging through a transparent window. **(B)** Example of intravital imaging setup based on 2-photon microscopy, including a pulsed laser with near-infrared (NIR) emission wavelengths and photomultipliers (PMT) for fluorescence detection. **(C)** 4D videos (3D z-stacks over time) capturing cell motility are acquired and visualized on a computer. Cells are tracked (white lines) to compute metrics such as speed, directionality (dir), and plotting of tracks with a common origin (PTCO).

The next step to perform a MP-IVM protocol is to select the proper surgical model, to enable the exposure and immobilization of the targeted organ (**Figure 1A**) (8, 9, 55). Although this typically requires minimally invasive surgery, more advanced surgical setups can be employed for long-term imaging of internal organs, including gut (56), brain and spinal cord (57), primary tumors and metastasis (58, 59) amongst others (60–63).

### Image Acquisition

Once surgery is completed, the anesthetized animal is transported to the microscope where image acquisition is performed. The fluorophores present in the sample are excited, and the resulting emitted fluorescence is acquired. A number of microscopy platforms are available for intravital imaging of the immune system (11, 64). Amongst these, multiphoton microscopy (MP-IVM) allows for deeper tissue penetration (by reducing scattering and autofluorescence) and prolonged acquisition time (by significantly reducing photodamage) (65, 66). This is achieved by employing a pulsed laser that emits excitation photons in the near-infrared range (NIR). The simultaneous absorption of multiple photons by a single fluorophore leads to the emission of one photon with higher energy. Finally, emitted photons are collected with detectors such as high-sensitivity photomultipliers (**Figure 1B**) (67).

4D imaging data (time lapses of 3D z-stacks) are obtained at different time points by sliding the excitation point throughout the sample on a focal plane and repeating this process by moving the focal plane along the z-axis. One drawback of this process is the reduced acquisition speed of MP-IVM. Conversely, other technologies such as resonant scanners or spinning disk confocal microscopy may be employed to capture rapid biological processes such as short-lived interactions or morphological changes (68).

### Data Analysis

The standard pipeline to analyze IVM videos consists of tracking the cells in the field of view, then computing motility measures from the cell trajectories (**Figure 1C**) (69, 70). Computer vision stands as a promising approach to automatically performing cell tracking (71). However, to date, a series of limitations hamper the accuracy of state-of-the-art automatic tracking algorithms when applied to immune cells observed *via* MP-IVM. For example, the high plasticity of the immune cells might yield to double tracking errors (69). Additionally, the high cell density associated with biological processes such as swarm formation hinder the distinction of individual cells. Lastly, technical artifacts introduced by the intravital imaging, such as varying signal-to-noise ratio across space and time, might affect the overall experimental readout (72, 73).

Therefore, to obtain insightful results, manual tracking and editing of automatically generated tracks are still required. Indeed, manual tracking significantly minimizes tracking errors and improves the accuracy of motility measures used to quantify cell migration and interaction (13). However, these procedures are time-consuming and prone to bias from each individual researcher.

### Common Measures of Cell Motility

A variety of measures formerly used to study particle dynamics in physics have been adopted by image analysts to study cell motility in different experimental setups, including intravital imaging (69, 70). Amongst these, speed and confinement ratio (also known as directionality, or meandering index) are the most common parameters when performing MP-IVM analysis of immune cells. Speed is defined as the ratio between the track length and the track duration, while the confinement ratio is defined as the distance between the first and the last point of the trajectory (displacement) divided by the total length of the track followed by a cell. This parameter tends to 1 for straight tracks, but decreases to 0 for circular tracks.

The aforementioned measures can be computed either on entire tracks (*track-based*) or on track fragments (*step-based)* (51). Track-based measures describe the overall motility of a cell for the entire period of observation. An important limitation in the application of these measures is that tracking errors can compromise the readout. Additionally, a cell whose behavior varies over time is represented by a single average value, yielding to an information loss. By contrast, step-based measures are computed amongst adjacent time points only, or on a temporal window, limiting the temporal propagation of errors. Moreover, step-based measures further allow the quantification of instantaneous changes in cell behavior, which may occur over time, rather than taking an average value of the entire track. If cells cannot be tracked for long periods of time, a step-based measure may represent the only possible choice for quantification.

More advanced measurements to evaluate the directionality of cells have also been defined. For example, the mean squared displacement analysis (MSD) evaluates the diffusivity of a particle by comparing it with the expected motion of a random walk. This measure can be represented by plotting the squared displacement over consecutive time steps, resulting in a straight line for a randomly diffusive process. Conversely, plots above this line refer to super diffusive, or directed, processes, while plots below it indicate a confined motion (74). The motility coefficient, expressed in µm^2^/min, is a diffusivity measure derived from the MSD (70). This coefficient considers the square of the cell displacement over time, which can be inferred as the slope of the MSD plot and can be used to compare migratory modes of different cells. In addition, the distribution of the turning angles can be evaluated to assess how much a cell deviates from its previous path. Following this analysis, narrow distribution centered on small angles are indicative of straight trajectories (75).

## Actions Performed by Individual Cells

### Patrolling

**Figure 2:**
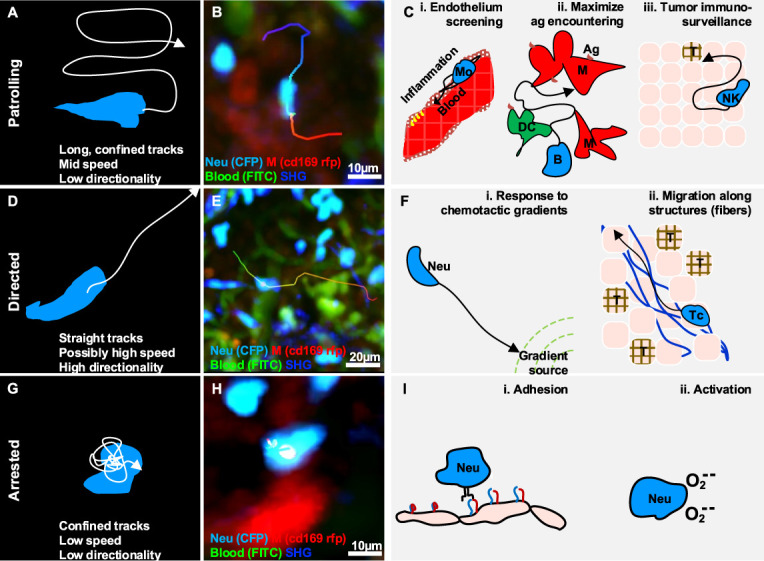
Gallery of actions displayed by individual immune cells. **(A)** Illustration of a patrolling cell, with the characteristic long track in a confined area, which is associated with mid-speed and low directionality (high confinement). **(B)** MP-IVM micrograph showing a patrolling neutrophil (light blue) migrating between macrophages (red) in the subcapsular sinus of a lymph node following infection. **(C)** Illustration of biological cases of patrolling behavior, including **(i)** a monocyte (Mo) screening the endothelium of blood vessels, **(ii)** a B cell surveying antigen-presenting cells in the lymph nodes (M: macrophages, DC: dendritic cells), and **(iii)** a natural killer (NK) cell during immune-surveillance in tumor microenvironments (T). **(D)** Illustration of a cell migrating directionally, with the characteristic straight tracks associated with high directionality and possibly high speed. **(E)** MP-IVM micrograph showing a neutrophil (light blue) exhibiting directed migration towards the subcapsular sinus area of a lymph node following infection. **(F)** Illustration of biological cases of directed migration including **(i)** a neutrophil (Neu) directed towards the source of a chemotactic gradient, and **(ii)** a T cell (Tc) moving with directed migration while following collagen fibers (blue structures) in the tumor microenvironment (T). **(G)** Illustration of an arrested cell with the characteristic folded track, which is associated with a low speed and high confinement. **(H)** MP-IVM micrograph showing a neutrophil (light blue) arresting in the proximity of a macrophage (red) in the subcapsular sinus area of a lymph node following infection. **(I)** Illustration of biological cases of arresting including **(i)** a neutrophil (Neu) during an adhesive interaction with an epithelial cell layer, and **(ii)** a neutrophil arresting during the production of reactive oxygen species.

Patrolling, also referred as scanning (76) or stochastic migration (46, 77), is an action associated with random-like movement characterized by long tracks in a confined area, which results in low directionality (**Figures 2A, B**) (19). The speed of patrolling cells varies according to the cell type, conditions, and anatomical site. For instance, monocytes exhibited a speed of 36 µm/min in the endothelium of carotid arteries and 9 µm/min in the mesenteric venules (52), while B cells exhibited a speed of 6 um/min in the lymph node follicles.

Patrolling cells are found in different biological processes occurring both at steady state and under inflammatory conditions.

#### Maximization of antigen encountering in steady state conditions

Patrolling cells are capable of monitoring large areas and promptly responding to specific antigens. For example, monocytes display a patrolling behavior while monitoring the endothelium of blood vessels (**Figures 2C, i**) (19). Upon activation, these cells promote the recruitment of immune cells locally *via* paracrine secretion of proinflammatory cytokines (16, 19, 20, 34, 52), and transient interactions (21). Similarly, a population of neutrophils were described with a patrolling behavior within the lumen of blood vessels. This was and associated with an increased capacity of these cells for being recruited to the inflammation site (78, 79). More recently, tissue-resident eosinophils have also been reported to display a patrolling behavior in different organs (33, 40).

In the LN, patrolling B cells continuously survey subcapsular macrophages and follicular dendritic cells in order to identify antigens that are either presented on a cell surface or suspended in the environment (80). Moreover, within the germinal centers (GC), patrolling B cells exhibited a probing, dendritic morphology that conferred them a larger surface area and therefore a greater opportunity for antigen encountering (**Figures 2C, ii**) (80). In addition, patrolling of T cells was also reported as a strategy to maximize the encountering of antigen presenting cells (APCs) (46, 77, 81) and avoid obstacles in densely packed microenvironments (82). Finally, Bajenoff and colleagues reported that the apparently random movement associated with the patrolling of T cells in the LN is indeed reflecting the complex network of fibroblastic reticular cells (47).

#### Patrolling under inflammatory conditions

NK cells were reported to maintain a patrolling behavior during priming (26), and while searching for cognate targets and transformed cells (**Figure 2C, iii**) (24), suggesting that the patrolling pattern is an efficient strategy for sensing and integrating cytokine signals under inflammatory conditions (26). Similarly, T cells displayed a patrolling behavior in the LN, to integrate signals from multiple APCs. Upon the encountering of APCs this behavior was maintained if the affinity was low, or switched to the formation of local clusters in case of high affinity (83).

Moreover, within the tumor microenvironment, patrolling monocytes were also associated with immune surveillance, promptly detecting tumor material, establishing interactions with metastasizing cells, and promoting recruitment and activation of natural killer (NK) cells in lung carcinoma (49).

### Directed Migration

Directed migration is associated with cells displacing along straight trajectories. These cells typically exhibit long tracks with high confinement ratio and possibly high speed (**Figures 2D, E**) depending on the cell type, the conditions, and the microenvironment.

In inflammatory contexts, cells undergo directional migration in response to chemotactic cues and inflammatory signals, as well as when influenced by anatomical structures. Generally, directional migration is described as a strategy to rapidly reach a specific target, which also plays important roles in recruitment, tissue repair, cleaning, and antigen presentation (84, 85). Amongst the different biological context where cells display this action we can find:

#### Response to Chemotactic Gradients

One of the best-characterized processes associated with directional migration is chemotaxis, which involves the polarization and displacement of cells towards the source of a chemotactic gradient (**Figures 2F, i**). For instance, neutrophils perform directed migration towards injured, infected, or inflamed areas (35, 37, 44, 86, 87), where their presence is relevant for tissue repairing, microbial clearing (88), amplification of the inflammatory response (89), and shaping of the adaptive immune response (90). In addition, macrophages perform directed migration in interstitial tissue in response to bacterial infection or tissue injury (91).

#### Influence of Anatomical Structures on the Directed Migration

Tissue architecture can influence cell movements, conferring properties of directed migration. The most compelling example is the transportation of cells *via* the bloodstream (92, 93). More recently, transportation of immune cells *via* lymphatics (94, 95) was also reported and associated with a strategy for rapidly reaching lymphoid tissues (84). Moreover, the architecture of the LN was reported to influence the recruitment of B and T cells, which displayed directional migration to relocate precisely in their respective areas (96). Finally, directed migration of immune cells was also associated with the architecture of tumor microenvironments. For instance, CD8+ T cells exhibited a directed migration pattern along collagen fibers in a model of ovarian carcinoma (50) (**Figures 2F, ii**).

### Arresting

Arresting is an action associated with cells that typically display confined trajectories and a speed below a predefined threshold (96) (**Figures 2G, H**). However, the migration of immune cells typically involves alternating cycles of “stop-and-go” (97). Hence, to define a cell as arrested, we consider that it should be tracked for longer than the duration of the stop-and-go cycle.

During the inflammatory process, motile cells change their behavior to arresting in order to perform a variety of functions, including signaling, killing, and activation.

#### Cell Activation and Signaling

Effective intracellular communication requires arresting. Notably, both B cells and T cells undergo an arresting phase prior to interacting with DC during priming (98). This step is essential to maximize the contact duration and to induce signaling.

In neutrophils, arresting was associated with the oxidative burst (38), a state in which reactive oxygen species are generated. This occurs both during phagocytosis and in response to soluble antigens. In contrast, Beuneu and colleagues (26) reported that NK cells do not arrest while being activated by DC. However, NK cells were reported to arrest in the medullary part of the LN (99) following influenza vaccination. Although the arrested NK cells were forming stable contacts with macrophages, this behavior was not associated with NK-mediated lysis. Therefore, it may suggest an alternative activation pattern.

#### Killing

The formation of stable contacts between a cytotoxic cell and its target is one of the best-characterized biological processes during which cytotoxic cells arrest. For instance, CD8^+^ T cells arrest during the formation of the cytotoxic synapses with target cells and resume their migration after killing the target (48, 100).

#### Adhesive Interactions During Recruitment

During recruitment from the blood stream, several types of leukocytes form adhesive interactions with stromal cells, leading to a decrease in motility and eventually to their arrest (22, 96) (**Figure 2I**). This process has been extensively revised (4, 101) and coincides with findings showing, that T cells interacting with lymphatic capillaries were commonly arrested (18).

## Actions Performed by Two or More Cells

Studies of cell migration have typically been performed by quantifying the motility of individual cells. Collective migration patterns, meanwhile, are more difficult to interpret (69) but they remain necessary for understanding complex biological processes such as inflammation. Indeed, Mayor and colleagues argue that considering cells as part of supracellular entities allows the quantification of migration at a higher scale (102).

### Contact Formation

**Figure 3:**
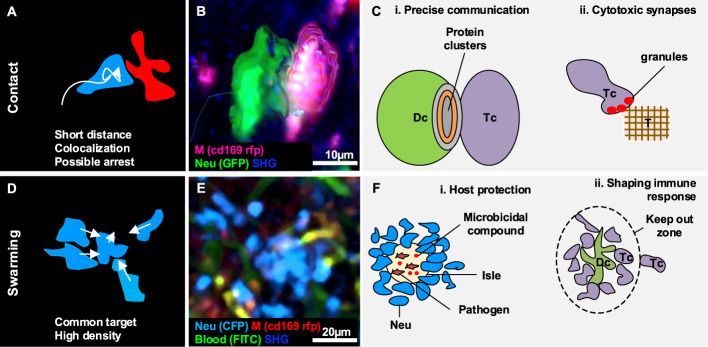
Gallery of actions displayed by two or a collectivity of cells. **(A)** Illustration of the morphodynamics of contact formation between two cells, characterized by a low distance and the possible overlap of colors. **(B)** MP-IVM micrograph showing a neutrophil (green) establishing contact with a macrophage (violet). 3D reconstructions are shown to highlight the shape of the cells during the formation of the contacts. **(C)** Illustration of biological cases of contact formation including **(i)** a T cell (Tc) forming an immunological synapse with a dendritic cell (Dc) with a cluster of proteins in the contact area, and **(ii)** a T cell (Tc) accumulating cytotoxic granules in contact with a tumor cell (T). **(D)** Illustration of the morphodynamics of swarm formation, characterized by cells moving towards a common target, resulting in the accumulation of cells in a confined area (high density). **(E)** MP-IVM micrograph showing a neutrophil swarm (light blue) following infection in the subcapsular area of a lymph node. **(F)** Illustration of biological cases including **(i)** a swarm of neutrophil (Neu) to contain pathogens in an isle enriched with microbicidal compounds, and **(ii)** a swarm of T cell (Tc) accumulating around an antigen-presenting dendritic cell (Dc) to prevent the other Tc from interacting with the Dc.

Contact formation is an action characterized by the absence of space between two or more cells (103) (**Figures 3A, B**). Indeed, during contact formation, the distance between membranes of cells decreases up to a distance of 15 nm to 100 nm (104). Cells forming contacts may exhibit an arrested behavior or maintain a patrolling behavior according to the duration and the type of contact.

Cellular contacts are a form of cell-to-cell communication that enables the formation of clusters between the proteins on the surface of distinct cells (**Figures 3A**–**C**) (105, 106), and the delivery of highly localized signals (40). Although contacts are continuously formed and disrupted between migratory and resident cells in physiological conditions, certain contacts of immune cells are important for the inflammatory processes (99, 107) due to their involvement in the modulation of the immune response.

#### Immunological Synapses

One of the best-characterized cases of contact formation between immune cells is the immune synapse that occurs between DC and T cells (**Figures 3C, i**). This process can occur either in lymphoid organs such as the LN (28), or non-lymphoid organs such as the lymphatic capillaries of the ear skin (18), and is pivotal for immunity and tolerance (29). DC play a crucial role in initiating the immune cell response as they scan the surrounding environment in search of antigens to capture and present to naive T cells (108). The interaction between T cells and DC follow a series of steps characterized by a varying contact duration. At first, T cells engage many short-lived contacts with the surrounding DC, reducing their overall motility due to the multiple interactions (25). Upon successful encountering of a DC presenting the antigen specific for the T cell receptor, long-term stable contacts occur, and T cells remain arrested, which leads to their activation. Finally, the T cell recover its motility and proliferate. This process has been observed in an OT-I model, where a comparison between antigen-specific CD8^+^ T cells and polyclonal CD8^+^ T cells revealed that antigen-specific cells significantly decreased their speed in response to the formation of stable interactions with DC (109). By contrast, polyclonal CD8^+^ T cells maintained a constant speed (109). This finding is in agreement with the T-DC model, where different phases of the T cell-DC interaction were associated with different contact durations (28), and highlights the importance of contact duration for efficient cell activation (30).

Moreover, some studies reported that NK cells maintained a motile behavior during the formation of short-term (1 – 3 min) contacts with DC, by recognizing cytokines on the surface of DC in addition to soluble signals (23, 43). This suggested an efficient strategy to sense and integrate cytokine signals from multiple DC (23). However, other *in vivo* studies have reported the formation of stable contacts with macrophages during the activation of these cells (99), in accordance with previous studies performed *in vitro* (110, 111).

#### Cytotoxic Synapses and Lysis

Cytotoxic T leukocytes (CTL) can establish cytotoxic synapses with target cells, which eventually leads to the lysis of the target (112). Cytotoxic synapses formed by CD8^+^ T cells (**Figures 3C, ii**), rely on a shared molecular mechanism with CD4^+^ T cell immunological synapses (112). However, CD8^+^ T cell synapses appear to be more stable and efficient in killing the target (113). Two known killing mechanisms involve the binding of Fas death ligand to Fas death receptor, resulting in the induction of apoptotic death by caspase activation (114). The second mechanism involves calcium-dependent release of perforin and granzymes, yielding to the activation of alternative apoptotic pathways (114). The latter mechanism was reported to be faster since it does not require specific receptors to be activated (112). Common targets of CTL are virus-infected or transformed cells. Moreover, CTL killing efficiency was reported to be affected by the affinity for its ligand (25, 112).

NK cells are also able to form contacts to lyse target cells through degranulation of lytic enzymes. Within the LN, NK has been observed to form contacts with B cells to eliminate major histocompatibility complexes mismatched targets (24). Additionally, in the context of tumor microenvironment, NK-mediated lysis was reported to occur either by establishing contacts of long duration with a single NK or *via* multiple short contacts with several NK (26).

### Swarming

Swarming is an action that involves a collectivity of cells clustering in a defined space or moving towards a common target in a coordinated manner, giving rise to a swarm (**Figures 3D, E**) (42). Swarms have been classified according to their size and duration (42). Transient swarms with fewer than 150 cells are reported to last up to 40 minutes. Larger swarms can include more than 300 cells and can persist for hours (42).

The swarming process has been primarily described in neutrophils, which form cell aggregates in inflamed and injured tissues. Notably, duration and swarm size were positively correlated with the severity of the tissue damage or infection, with extended lesions massively recruiting neutrophils involved in swarms that persisted for days (32, 115). Cell death, known to induce recruitment of phagocytic cells (51, 116, 117), is regarded as one of the triggers of swarming.

Swarming is associated with two key biological functions, host protection and tissue remodeling.

#### Host Protection

Swarm formation was reported in infection models as a strategy to contain pathogens and protect the host (118). To this end, swarms lead to the confinement of pathogens in isles where microbicidal compounds concentrate (**Figures 3F, i**) (115). Accordingly, neutrophil swarming was observed to contain bacteria spread (39) and limit the growth of fungi *in vivo (* 119). Eosinophils were also observed performing swarms throughout the parenchyma in the lungs in different infection models. Amongst these, during parasitic infections, swarms of eosinophils were maintained for several days (120).

#### Tissue Remodeling and Shaping the Immune Response

Neutrophil swarming was also reported in the context of sterile inflammation. Sterile photo burning (87) and needle damage (44) caused neutrophils to form abrupt and long-lasting clusters of large dimensions, suggesting a role in tissue remodeling and repair.

Additionally, the formation of swarms can alter the cellular structure of immune organs. For instance, swarms formed by neutrophils were reported to disrupt the network of resident SCS macrophages in parasitic infection models (41, 115, 121). Considering that SCS macrophages are important for containing the spread of pathogens (122, 123) and for activating the adaptive immunity (86, 122), the alteration of this cell layer by swarms might influence the overall immune response. Interestingly, other cell types, such as NK cells, were also observed to form swarms in the SCS area of the LN and to interact with resident CD11b^+^ cells. The accumulation of NK cells in the SCS area was linked to the function of promoting self-activation by the encountering of specific APC (124). Other cell types such as T cells were reported to form swarms around APC following immunization. Since most of the interactions in the swarms were maintained over time (108), it has been proposed that swarms may keep newly arrived T cells at the boundaries of the swarm, limiting their interaction with DC (**Figures 3F, ii**) (108). Finally, swarming of invariant natural killer T cells was associated with a reduced level of fibrosis in a model of steatohepatitis (17), suggesting a further role of swarming in tissue remodeling under inflammatory conditions.

## Methods to Detect and Quantify Cell Actions

Due to the difficulties associated with the processing and quantification of IVM movies (125), computational methods have become essential for the analysis of cell motility *in vivo*. A variety of software and methods were applied to detect and quantify cell actions. A summary of these methods, including how to use them and how to interpret the computed numerical values is presented in **Table 2**.

**Table 2:** Software and tools to quantify cell actions.

Action	Tools	How to use	How to interpret	Requires surfaces	Requires tracking
**Patrolling**	Imaris	After having tracked each cell, use the Filter tool to select tracks according to Track Length and Track Straightness.	High Track Length, and mid–low Track Straightness are indicative of patrolling	no	Yes
	Icy, QuantEV	Launch the QuantEV plugin (track processor) and select tracks according to the confinement ratio distribution	A confinement ratio distribution skewed towards the right indicates patrolling	no	yes
	Fiji. Trajectory classifier	Run the Trajectory classifier for TrackMate plugin, analyze the tracks	Patrolling cells are typically classified as “subdiffusive”.	no	yes
**Directed**	Microsoft Excel, Matlab, Imaris	Import into Microsoft Excel, Matlab, or a similar program the standard track measures, such as Track Duration and Track Straightness from Imaris. Exclude short tracks (i.e., < 300s) or add a rule to compute normalized Track Straightness.	Track Straightness is close to 1 indicates directed migration	optional	yes
	Icy, QuantEV	Launch the QuantEV plugin (track processor) and select tracks whose	A confinement ratio distribution skewed towards the left indicates directed migration	no	yes
	Fiji, Trajectory classifier	Run the Trajectory classifier for TrackMate plugin, analyze the tracks.	Directed cells are typically classified as “directed/active motion”.	no	yes
**Arresting**	Imaris, Arrest Coefficient XT	Select the cells of interest, launch the plugin, and define a speed threshold to consider a cell arrested. The plugin computes the arrest coefficient and counts the number of stops for each cell.	Values of the arrest coefficient close to 1 indicate arresting	optional	yes
	Icy, QuantEV	Launch the QuantEV plugin (track processor) and select tracks whose lifetime is sufficiently high.	Total path length of arrested cells is typically low.	no	yes
**Contact formation**	Imaris, Kiss and Run XT	Launch the plugin, define a distance threshold to detect a contact (i.e., 2 µm) and select two surfaces (i.e., two types of cells) to compute contact number and duration for each single cell.	The plugin automatically reports the number and the duration of contacts which can be used to discriminate between short- and long-lived interactions	yes	optional
	Imaris, Colocalization, Matlab	To detect contacts between cells of different color, launch the Coloc functionality to create an imaging channel specific to the contacts. Create a surface on this new channel and export the number of surfaces to count contacts. Smoothing can be applied to enhance contact detection with minimal overlap.	Contacts are associated with regions having a high brightness intensity in the created colocalization channel	no	no
**Swarming**	Matlab/R, etc.	Import cell tracks, compute the distance over time vs. a common target.	If multiple cells display a reduction of the distance over time towards a common target, this might recall a swarming behavior.	no	yes
	Matlab/R, etc.	Import cell tracks and compute a density map based on the emitted fluorescence, or a velocity map based on optical flow	Swarming is associated with regions having high density and convergent velocity tensors	no	yes
	Imaris	Reconstruct a surface on all the cell of interest with large smoothing (> expected cell diameter), divide the surface volume by the typical cell volume to overestimate cells in the swarm, and apply smoothing to fill gaps.	Swarming is associated with large areas or volumes of the reconstructed surfaces. A growing behavior can be inferred by plotting the surface area or volume over time	yes	no

### Quantification of Patrolling Cells

To quantify the patrolling behavior, coefficients that evaluate the displacement over time, such as the directionality and the motility index are typically used. These coefficients are larger than the ones displayed by arrested cells, but lower than the values displayed by directional cells (18, 126, 127). Additionally, the previously mentioned MSD analysis can be used to distinguish patrolling cells from arrested or directed cells, as they display a random-like migratory pattern. However, several studies demonstrated that the movement of cells *in vivo* is not stochastic, but rather influenced by alternative parameters such as the interaction with stromal cells, amongst others (70). Therefore, we suggest to complement the MSD analysis with other parameters, such as the angle or speed distribution, which would provide additional insights on the migratory mode.

### Quantification of Directed Cells

Directional migration can be inferred by plotting the trajectories of the analyzed cells with a common origin, resulting in tracks with a strong preferential migration direction (45, 51, 63). More quantitatively, one of the parameters that better characterizes directed migration is the confinement ratio, which presents high values for highly directional cells (69). However, the confinement ratio of different tracks is comparable only if they have similar track durations. Otherwise, normalizing the trajectories for their duration is often required. Another measure typically used for predicting directed migration is the distribution of the turning angles (36). Following this analysis, a skewing toward small angles would indicate that a cell trajectory does not deviate abruptly from its established path. In addition, the MSD analysis could also indicate directional migration recalling super diffusivity (36, 44).

When analyzing the overall motility of a cell population, directional migration can be inferred by evaluating the distance over time of all the cells with respect to a reference point or region. The chosen reference point should ideally represent a common target (32, 41, 51, 115) towards which the distance decreases or increases.

### Quantification of Arresting Cells

An arrested cell is typically detected by evaluating the arrest coefficient (69). This coefficient measures the amount of time in which a cell migrates with a speed below a defined threshold (typically 2 µm/min) (31). However, the value of the arrest coefficient depends on the track duration. Therefore, tracks (or track fragments) with similar duration should be compared; otherwise, normalization strategies are required for comparative studies (i.e., dividing the arrest coefficient by the track duration). The confinement ratio used to predict the directionality of a trajectory is also used to detect arrested cells, which typically display low values (31).

### Quantification of Contacts

Contacts are typically detected by evaluating the distance between cells. Such a distance can be computed either between the centroids of the cells (75), or reconstructed surfaces (i.e. between the closest points of two cells) (13). In the first case, a contact is detected when the distance is less than a threshold, which is equal to the expected cell diameter. However, errors may be introduced when cells with a non-convex shape are analyzed. In the second case, the distance threshold is preferably small, up to the spatial resolution of the microscope (i.e., 1 µm). This allows to detect contacts between cells of arbitrary shapes. However, the distance between the membranes of two cells forming a contact is on average lower than the spatial resolution of fluorescent microscopes used in intravital imaging (0.2 µm – 0.3 µm) (128). Moreover, an accurate reconstruction of cell surfaces might be hampered by the presence of cell-to-cell contacts themselves (13). For these reasons, cell-to-cell contacts are still annotated manually (18). More robust approaches inferred contact formation from time series, such as the trajectories of the individual cells or the changes in cell speed (27). Moreover, spatial colocalization of two distinct fluorophores can be used to highlight overlapping cells without the need for surface reconstruction (27) nor the computation of spatial distances between cells.

Contact dynamics can be quantified by computing for each cell the contact duration and the number of contacts. This ultimately allows one to distinguish between transient and long-lasting contacts.

### Quantification of Cell Swarms

To quantify swarm dynamics in the case of localized tissue damage or infection, the distance between the affected region and each cell at different time points can be computed. Cells whose distance over time falls below a defined threshold are considered part of a forming swarm (32, 41, 44, 51, 63).

Alternatively, when the swarm coordinates are not known, the increase of fluorescence intensity over time in different areas can be computed. In turn, surface and volumetric reconstruction enable the monitoring of the swarm growth over time by encompassing the fluorescence intensity emitted by the forming swarm (41, 45, 51). This provides insights into the different stages of the process, including initiation, growth, stabilization (41) and whether the swarm is transient or persistent (119). Furthermore, dividing the measured surface or volume by the mean volume or area of cells leads to an estimate of the number of swarming cells (41).

Swarming can be also inferred from the trajectories and speed of cells. Indeed, color coding the cell trajectories for their instantaneous cell speed can help to locate transient and persistent swarms (39). Similarly, representing a heatmap of the cell velocities and densities generates a spatiotemporal visualization that accounts for both migratory and clustering dynamics (38).

## Concluding Remarks

In line with the computer vision community, we considered distinct motility patterns displayed by cells as elementary actions (129, 130), which are the building blocks of several biological processes. This approach is relevant to dissecting the complex dynamics of inflammation, as it provides a link between identifiable morpho-phenotypes and the underlying cellular function. Moreover, by detecting the occurrence of each action over time, it is possible to quantify the dynamic behavior of immune cells in response to different stimuli.

Another advantage of decomposing cell motility in elementary actions is that these can be quantified from tracks of short duration (tracklets) or short image sequences using instantaneous measures. However, a longer imaging time can lead to more accurate results for actions such as swarming, which can persist for up to several hours.

In this review, the visual approach adopted to classify each action further aims to facilitate the interpretation of intravital microscopy data for immunologists and imaging specialists. The described measurements and definitions are provided to help researchers in differentiating between distinct cellular actions from a motility perspective. These are, however, intended only as guidelines rather than absolute discriminatory factors, as no consensus definition and numerical characterization exists thus far. In fact, to identify cell actions, it might be necessary to adopt a gating strategy that considers the combination of several motility parameters (51). Future advancements in this direction will require further characterization of cell motility based on the function, cell type, and organ.

In conclusion, the development of computer vision methods for cellular action recognition represents a promising methodology for deciphering biological processes occurring *in vivo* from imaging data.

## Inclusion Criteria

This review includes studies that reported specific motility patterns of immune cells, which were observed under inflammatory conditions *in vivo*. The included imaging modalities were MP-IVM, spinning disk, laser scanning confocal, and epifluorescence microscopy. All the definitions of cell actions used in this work were inferred from the original studies. In most cases, the authors explicitly named the migratory patterns displayed by the imaged cells. Indeed, directed migration, arresting, contact formation and swarming (or clustering) are well-characterized processes that were typically referred to using a direct name. In these cases, we did not perform re-analysis of the data. In the case of patrolling instead, studies referred to it either with the same term, or a similar nomenclature (i.e., scanning, undirected migration, random migration), or provided measurements whose values were indicative of this motility pattern.

## Author Contributions

DP and AP reviewed the literature and wrote the manuscript. MT and RK corrected the manuscript. SG supervised and wrote the manuscript. All authors contributed to the article and approved the submitted version.

## Funding

This work was supported by the Swiss National Foundation (SNF) grants, 176124, SystemsX.ch (2013/124), Biolink (189699).

## Conflict of Interest

The authors declare that the research was conducted in the absence of any commercial or financial relationships that could be construed as a potential conflict of interest.

## Publisher’s Note

All claims expressed in this article are solely those of the authors and do not necessarily represent those of their affiliated organizations, or those of the publisher, the editors and the reviewers. Any product that may be evaluated in this article, or claim that may be made by its manufacturer, is not guaranteed or endorsed by the publisher.
